# Estimating the Cost of Care for Emergency Department Syncope Patients: Comparison of Three Models

**DOI:** 10.5811/westjem.2016.10.31171

**Published:** 2017-01-20

**Authors:** Marc A. Probst, John K. McConnell, Robert E. Weiss, Amber L. Laurie, Annick N. Yagapen, Michelle P. Lin, Jeffrey M. Caterino, Manish N. Shah, Benjamin C. Sun

**Affiliations:** * Icahn School of Medicine at Mount Sinai, Department of Emergency Medicine, New York, New York; † Oregon Heath & Science University, Center for Health Systems Effectiveness, Department of Emergency Medicine, Portland, Oregon; ‡ University of California, Los Angeles, Department of Biostatistics, Los Angeles, California; § Oregon Heath & Science University, Center for Policy and Research in Emergency Medicine, Portland, Oregon; ¶ The Ohio State University Wexner Medical Center, Department of Emergency Medicine, Columbus, Ohio; || University of Wisconsin, Madison, Department of Emergency Medicine, Madison, Wisconsin

## Abstract

**Introduction:**

We sought to compare three hospital cost-estimation models for patients undergoing evaluation for unexplained syncope using hospital cost data. Developing such a model would allow researchers to assess the value of novel clinical algorithms for syncope management.

**Methods:**

We collected complete health services data, including disposition, testing, and length of stay (LOS), on 67 adult patients (age 60 years and older) who presented to the emergency department (ED) with syncope at a single hospital. Patients were excluded if a serious medical condition was identified. We created three hospital cost-estimation models to estimate facility costs: V1, unadjusted Medicare payments for observation and/or hospital admission; V2: modified Medicare payment, prorated by LOS in calendar days; and V3: modified Medicare payment, prorated by LOS in hours. Total hospital costs included unadjusted Medicare payments for diagnostic testing and estimated facility costs. We plotted these estimates against actual cost data from the hospital finance department, and performed correlation and regression analyses.

**Results:**

Of the three models, V3 consistently outperformed the others with regard to correlation and goodness of fit. The Pearson correlation coefficient for V3 was 0.88 (95% confidence interval [CI] 0.81, 0.92) with an R-square value of 0.77 and a linear regression coefficient of 0.87 (95% CI 0.76, 0.99).

**Conclusion:**

Using basic health services data, it is possible to accurately estimate hospital costs for older adults undergoing a hospital-based evaluation for unexplained syncope. This methodology could help assess the potential economic impact of implementing novel clinical algorithms for ED syncope.

## INTRODUCTION

There is increasing pressure to improve the value of healthcare, defined as health outcomes per dollar spent.^1^ Hospital-based diagnostic evaluation has specifically received scrutiny and has been characterized as costly and overutilized.^2^ Syncope is responsible for over one million emergency department (ED) visits annually in the U.S. and is associated with substantial healthcare costs.^3^,^4^ Development of novel, evidence-based clinical algorithms, specifically for syncope, may improve the value of care.^5^

A major methodological challenge to evaluating the economic impact of clinical algorithms aimed at improving resource utilization is the absence of validated cost-estimation models.^6^ While there have been prior attempts to estimate aggregate ED costs, estimating patient-level hospital costs is difficult since patient-level financial data are not readily available for privacy and proprietary reasons. 6

The purpose of this brief research report was to compare three cost-estimation models with hospital cost data obtained from the hospital finance department. Our objective was to develop a model that could accurately predict the hospital costs of a diagnostic evaluation for older adults with unexplained syncope.

## METHODS

### Study Design

We used prospectively collected data on health services use among older adult patients who presented to the ED with syncope to compare three hospital cost-estimation models with actual hospital cost data. This study was approved by our institutional review board.

### Study Setting and Population

Our study sample consisted of older adults who presented to the ED at an urban, tertiary care, academic medical center (45,000 annual visits) with syncope. The data collection was part of a multicenter, prospective, observational study on syncope risk stratification (NCT01802398). Only our primary institution was used for the current study since this was the only hospital from which we were able to access hospital finance department data. Inclusion criteria were 1) age≥60 years, and 2) a complaint of syncope or near-syncope. Exclusion criteria were seizure, loss of consciousness after head trauma, ongoing confusion, intoxication, and intervention to restore consciousness. We also excluded patients from analysis if they had incomplete data or if a serious medical condition was identified in the ED or during the index hospitalization. Serious conditions included myocardial infarction, pulmonary embolism, gastrointestinal bleeding, stroke, cardiac arrhythmia, aortic dissection, severe structural heart disease, and other serious illnesses. The purpose of excluding patients with serious medical conditions was to estimate the diagnostic costs associated with unexplained syncope and *not* costs associated with the treatment of serious conditions.

### Key Outcome Measures

We obtained patient-level hospital cost data, i.e. resources spent to provide services, from the hospital finance department on the study sample. We did not analyze charges, which are often poorly related to costs, nor did we collect data on professional fees or patient co-pays since these were unavailable. Total hospital costs were obtained for the index hospital encounter. Hospital finance department cost estimates use a fully allocated operating expenses methodology, meaning that 100% of hospital operating expenses (both indirect and direct costs) are attributed to each patient charge item for a given time period. A cost per unit is the result of absorbing all direct and indirect expenses based on a combination of cost-weight methodologies. Cost per unit is multiplied by each charge-item quantity to calculate cost, which is then summarized at the patient, procedure, physician, and service line level.

Health service use was measured by chart review of medical records by trained, non-physician, research staff using a standardized data collection form. Assessment of inter-rater reliability on 10 charts demonstrated >95% concurrence on items that measured health service use. All charts with a potential serious outcome were reviewed by the senior author.

We used three different methods to estimate total costs. All three models were the sum of two components: 1) direct costs of tests, and 2) estimated facility costs. For all three models, the direct costs of tests was calculated by adding up the unadjusted payment rates for each individual test per Center for Medicare and Medicaid Ambulatory Payment Classifications (APC) payments (Appendix A).^7^ The three models differed only in the way in which facility costs were estimated.

For the first model (V1), “Unadjusted Medicare Payment,” published Medicare payments were used to estimate facility costs in the following manner: for patients discharged directly from the ED, we used evaluation and management (EM) Level 5 (APC code 616; $492.69) payment.^8^ For patients placed under observation status, we applied the Extended Assessment & Management (Observation) (APC code 8009) payment ($1,234.70). For patients with an inpatient admission, we applied the facility’s average Medicare payment for Diagnosis Related Group (DRG) “Syncope & Collapse” from 2013 ($5,575.16 at our institution). All admitted patients were assumed to have received a DRG classification for syncope (DRG code 312).

In the second model (V2), “Modified Medicare Payment, Prorated by LOS in Calendar Days,” we estimated facility costs in the following manner: for patients discharged directly from the ED, we used evaluation and management (EM) Level 5 (APC code 616; $492.69) payment, as in model V1. For patients placed under observation status, or admitted to the hospital, we applied the same Observation APC code 8009 for each calendar day included in the total LOS. This model was proposed to explore whether length of stay (LOS) in days is a better proxy for cost than DRG or observation figures, as identical services can be delivered to a patient in either setting (in-patient or observation) and yet be billed differently.

In the third model (V3), “Modified Medicare Payment, Prorated by LOS in Hours,” we estimated facility costs in the following manner: for patients discharged directly from the ED, we used evaluation and management (EM) Level 5 (APC code 616; $492.69) payment, as in model V1 and V2. For patients placed under observation status or admitted to the hospital, we calculated an average hourly amount in this cohort based again on the Observation APC code 8009 payment and multiplied that average hourly amount by total LOS in hours. This model is potentially more accurate than V2 but does require more granular data (LOS in hours versus days).

### Data Analysis

We performed descriptive analyses of modeled costs. To assess the agreement between hospital cost data and modeled costs for each method, we generated scatter plots, calculated Pearson’s correlation coefficients and performed linear regression of direct costs on estimated costs. All analyses were performed in SAS 9.4 (Cary, NC, USA).

## RESULTS

### Characteristics of Study Subjects

We collected data on a convenience sample of 100 ED patients with syncope and age ≥60 years. Data collection occurred between April 29, 2013 – March 3, 2014. One patient was excluded due to incomplete data, and 32 were excluded due to a serious medical condition, leaving 67 patients for the final analysis. Included patients had a mean age of 73.4 years (range 60–98) and were 55% male (Appendix B).

### Main Results

Scatterplots of estimated costs compared to actual costs are presented in the figure in U.S. dollars. The primary analysis using raw data for the direct and estimated costs revealed that all three models (V1, V2, and V3) demonstrated strong to very strong Pearson’s correlation and linear regression coefficient with V3 performing the best (r =0.88 [95% CI 0.81, 0.92], regression coefficient 0.87 [95% CI 0.76–0.99]). The goodness of fit was also highest for V3 (0.77) (Table). The average estimated cost was $1,482, range [$347, $5,514]. The average actual cost was $1,486, range [$164, $4,893]. The intercorrelations between the three models can be found in Appendix A.

## DISCUSSION

We compared the performance of three cost-estimation models to predict the cost of care for unexplained syncope. One model, V3, consistently outperformed the other two models with respect to correlation with hospital finance data, which we used as the reference standard. By adding the individual costs of diagnostic tests (based on publicly available CMS data) and estimating facility costs using APC observation payments, prorated by LOS in hours, this model best predicted the total cost of care for patients with unexplained syncope. This model likely performed best because of two factors: 1) the inputs were more granular (hours versus days), thus leading to a more accurate estimation of the quantity of health services delivered; and 2) it removes the somewhat arbitrary payment differences between in-patient admission and observation stay, focusing instead on LOS as a proxy of the quantity of services delivered.

Developing a valid cost-estimation model would allow health services researchers to estimate costs associated with syncope without access to hospital proprietary information. Mounting pressures to contain healthcare costs have spurred researchers, administrators, and policymakers to devise and implement strategies to increase the value of care. Syncope was identified as one of the top conditions targeted by Medicare Recovery Audit contractors for repossession of medically unnecessary inpatient expenditures.^9^ Estimating the costs of syncope-related healthcare services at the patient level is a crucial step in being able to predict the economic effects of implementing novel syncope clinical algorithms.

## LIMITATIONS

Our study has certain limitations. First, our findings are from a small, single-site sample and should be validated in other settings. Second, our hospital finance department does not use strict activity-based costing, which is a highly resource intensive “gold standard” approach for cost estimation.^6^ However, hospital financial data appear to be a more accurate method of assessing costs than other available methods.^10^ We did not include professional fees or patient co-pays, both contributors to the overall costs of care, since these data were not available. However, hospital charges are generally the target of policies aimed at increasing healthcare value.

## CONCLUSION

In summary, we derived and compared three models for cost estimation that correlated with actual hospital costs. The most accurate model (V3) uses Medicare payments for diagnostic tests and requires hospital LOS in hours to estimate hospital costs for the diagnostic evaluation of syncope. This simple cost model could be a useful tool for investigators to assess the economic impact of novel clinical algorithms for syncope.

## Supplementary Information





## Figures and Tables

**Figure:**
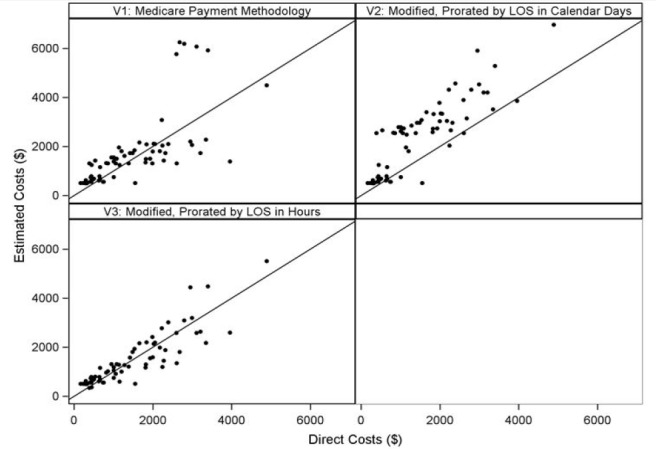
Scatter plots of estimated costs (V1, V2, V3) by direct costs for syncope care of older adults.

**Table:** Comparison of total cost estimation models (V1, V2, V3) versus actual hospital costs for syncope patients.

Method for estimating hospital costs	Pearson’s correlation coefficient (95% CI)	Linear regression coefficient (95% CI)	R-squared
V1: unadjusted Medicare payment	0.69 (0.54, 0.80)	0.51 (0.38, 0.64)	0.48
V2: modified Medicare payment, prorated by LOS in calendar days	0.86 (0.78, 0.91)	0.60 (0.52, 0.69)	0.75
V3: modified Medicare payment, prorated by LOS in hours	0.88 (0.81, 0.92)	0.87 (0.76, 0.99)	0.77

*CI*, confidence interval; *LOS*, length of stay.
