# Supplementary material for: Estimating the Cost of Care for Emergency Department Syncope Patients: Comparison of Three Models
Source: West J Emerg Med. 2017 Jan 20;18(2):253–7. doi: 10.5811/westjem.2016.10.31171 (PMC5305134; doi:10.5811/westjem.2016.10.31171)
Supplement: Supplementary file 1 [file wjem-18-253-s001.pdf]

Appendix A: Methods for estimating total hospital costs for patients presenting with syncope.

Total estimated costs = Direct costs of tests + Facility costs

Direct costs of tests: Sum of the unadjusted payment rates for tests per Center for Medicare and Medicaid Ambulatory Payment Classifications payments.

Facility costs: Three methods for estimating facility costs (V1, V2, V3):

	**Discharged**	**Admit to Observation**	**Admit to Inpatient**
**Source**	EM Level 5 APC 616	Extended Assessment & Management (Observation) APC 8009	DRG Syncope & Collapse, Average Medicare Payment
**Cost**	$492.69	$1,234.70	$5,575.16
**V1: Medicare Payment**	$492.69	$1,234.70	$5,575.16
**V2: Modified Medicare Payment, Prorated by LOS in Calendar Days**	$492.69	$1,234.70 * total LOS in calendar days	$1,234.70 * total LOS in calendar days
**V3: Modified Medicare Payment, Prorated by LOS in Hours**	$492.69	$1,234.70/mean total LOS for patients in observation * LOS in hours	$1,234.70/mean total LOS for patients in observation * LOS in hours

APC: Ambulatory Payment Classifications. DRG: Diagnosis Related Group. LOS: Length Of Stay.
