# Supplementary material for: Estimating the Cost of Care for Emergency Department Syncope Patients: Comparison of Three Models
Source: West J Emerg Med. 2017 Jan 20;18(2):253–7. doi: 10.5811/westjem.2016.10.31171 (PMC5305134; doi:10.5811/westjem.2016.10.31171)
Supplement: Supplementary file 2 [file wjem-18-253-s002.pdf]

Appendix B: Characteristics of patients presenting to the emergency department with syncope.

N=67	Number (percent)
**Age (in years, mean (range))**	73.4 (60-98)
**Male gender (%):**	37 (55.2%)
**Race:**	
White or Caucasian	60 (89.6%)
Black or African American	3 (4.5%)
Asian (Asian Indian, Filipino, Japanese, Other Asian)	1 (1.5%)
American Indian or Alaskan Native	1 (1.5%)
Refused	1 (1.5%)
Multiracial	1 (1.5%)
**Commonly Ordered Diagnostic Tests (%):**	
-Chest X-ray	46 (68.7%)
-CT Head	25 (37.3%)
-Rest Echocardiogram	20 (29.9%)
-Troponin	65 (97.0%)
-Carotid Ultrasound	9 (13.4%)
-Event Monitor	6 (9.0%)
**Disposition:**	
-Direct discharge from ED	21 (31.3%)
-Observation status	42 (62.7%)
-Admit to hospital	4 (6.0%)

**Length of stay (in hours):**	**Median (range)**
-All patients	18.9 (1.9-85.8)
-Direct discharge from ED	4.5 (1.9-7.3)
-Observation status	23.5 (4.0-72.3)
-Admit to hospital	37.2 (5.3-85.8)

ED: Emergency Department, CT: Computed Tomography.
